# Advanced Strategies for 3D Bioprinting of Tissue and Organ Analogs Using Alginate Hydrogel Bioinks

**DOI:** 10.3390/md19120708

**Published:** 2021-12-15

**Authors:** Qiqi Gao, Byoung-Soo Kim, Ge Gao

**Affiliations:** 1 Institute of Engineering Medicine, Beijing Institute of Technology, No. 5, South Street, Zhongguancun, Haidian District, Beijing 100081, China; 3120211985@bit.edu.cn; 2 School of Biomedical Convergence Engineering, Pusan National University, Yangsan 626841, Kyungnam, Korea; bskim7@pusan.ac.kr; 3 Department of Medical Technology, Beijing Institute of Technology, No. 5, South Street, Zhongguancun, Haidian District, Beijing 100081, China

**Keywords:** alginate hydrogel, bioink formulation, 3D bioprinting strategy, biomedical applications

## Abstract

Alginate is a natural polysaccharide that typically originates from various species of algae. Due to its low cost, good biocompatibility, and rapid ionic gelation, the alginate hydrogel has become a good option of bioink source for 3D bioprinting. However, the lack of cell adhesive moieties, erratic biodegradability, and poor printability are the critical limitations of alginate hydrogel bioink. This review discusses the pivotal properties of alginate hydrogel as a bioink for 3D bioprinting technologies. Afterward, a variety of advanced material formulations and biofabrication strategies that have recently been developed to overcome the drawbacks of alginate hydrogel bioink will be focused on. In addition, the applications of these advanced solutions for 3D bioprinting of tissue/organ mimicries such as regenerative implants and in vitro tissue models using alginate-based bioink will be systematically summarized.

## 1. Introduction

3D bioprinting is an automated biofabrication technique based on a layer-by-layer deposition process that can precisely orchestrate living cells, matrices, biomaterials, and molecules following a pre-determined model devised by computer-aid-design [1]. The burgeoning development of the 3D bioprinting technique has extensively stimulated the advances of regenerative medicine, individualized therapy, and customized biomedical instrument. For instance, to tissue-engineer artificial tissue substitutes, 3D bioprinted structures may be used as an instructive scaffold or even directly maturated into a functional tissue-equivalent when living cells are involved during the printing process [2]. Besides tissue regeneration and repair, 3D bioprinting is also capable of constructing advanced in vitro tissue models, such as organ-on-a-chip and organoids, that can be either utilized as drug screening platforms for analyzing pharmaceutic safety and efficacy in patient-specific conditions or applied as an alternative tool of animal models for the interpretation of disease pathophysiology [3,4]. Therefore, 3D bioprinting has been recognized as one of the most promising biofabrication techniques over the past decade.

As a key element of 3D bioprinting, the term bioink was originally introduced with the advent of the organ printing concept in 2003 [5]. Bioinks not only escort the encapsulated living cells and molecules to build complex 3D structures but also provide a microenvironment for regulating cell activities and ECM remodeling [6]. Hence, appropriate physical and biological properties are the basic requirement for an ideal bioink. The physical properties of a bioink include (1) tunable viscosity that can be adapted to different 3D bioprinting techniques, (2) shear-thinning rheological behavior which helps to protect cell viability during the printing process, (3) acceptable printability enabling successfully fabrication of complex constructs, (4) mild gelation condition for minimizing cell damages, and (5) proper mechanical properties applicable for biomedical application scenarios. In the view of biological properties, on the other hand, the bioink should be (1) non-immunogenic when used to build tissue and organ implants, (2) bio-instructive for promoting the adhesion and migration of residing cells, (3) biodegradable allowing for ECM remodeling and controlled release of payloads. To date, a variety of natural biomaterials (e.g., ECM proteins, polysaccharides, glycosaminoglycans) and synthetic polymers have been formulated as bioinks [7]. However, none of these biomaterials can fully match the abovementioned requirements.

Among the currently available bioink candidates, alginate has received extensive attention. As a low-cost natural polysaccharide normally derived from brown algae, alginate possesses multiple essential advantages, including non-immunogenic, biodegradable, and non-cytotoxicity features, as well as the rapid and cell-friendly gelation characteristic [8]. For these reasons, alginate has been widely used as a biomaterial for tissue engineering and regenerative medicine (e.g., wound healing [9] and bone regeneration [10]). Despite its unique properties, numerous critical drawbacks limit its application as a bioink for 3D bioprinting. First, although the viscosity of alginate pre-gel is tunable as the concentration varies, its poor printability leads to the difficulty of direct 3D bioprinting of complex structures [11]. Besides, this material is bioinert due to the absence of cell-adhesive motifs and thus may lead to anoikis of embedded cells. Moreover, the alginate hydrogel is dissolvable upon interactions between monovalent cations existing in reagents and alginate blocks, which is detrimental to the long-term structural and mechanical stability of the printed structures for in vitro tissue modeling [12]. Furthermore, the non-bonded alginate polymers never degrade via enzymatic activity in mammalian hosts; therefore, the implants composed of alginate hydrogel usually suffer from erratic biodegradation kinetics [13]. Hence, to utilize alginate as a versatile bioink, it is indispensable to improve several performances of this material, such as enhancing printability, promoting cellular affinity, and controlling biodegradation manner and kinetics.

**Figure 1:**
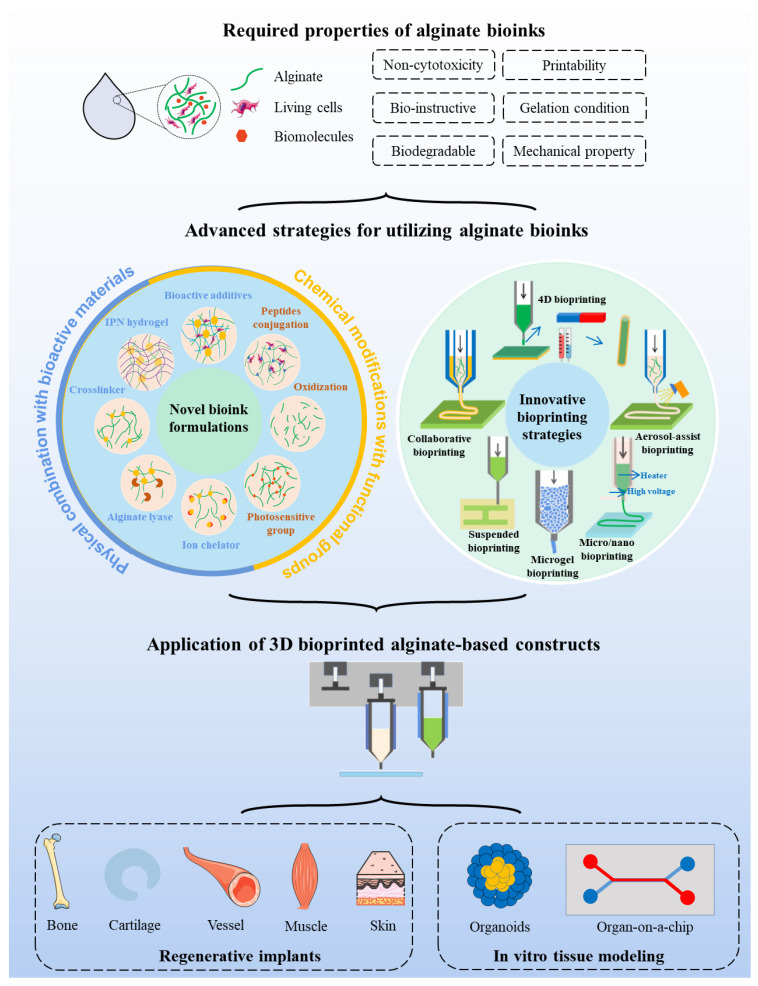
Schematic illustration representing advanced strategies for improving the performances of alginate bioinks, as well as their applications in 3D bioprinting of regenerative implants and in vitro tissue models. This figure was prepared using a template on the Sevier medical art website (http://www.sevier.fr/sevier-medical-art, accessed on 25 November 2021).

As illustrated in a schematic view (Figure 1), this review provides an overview of the current strategies that attempt to overcome the limitations when alginate is selected as a bioink for 3D bioprinting. The article will explain the working principle of prevalent 3D bioprinting techniques and discuss the pivotal properties of bioinks. Afterward, a variety of advanced material formulations and biofabrication strategies that are recently developed to break through the bottleneck of alginate bioink will be focused upon. In addition, the applications of these advanced solutions for 3D bioprinting of regenerative implants and in vitro tissue/organ models using alginate bioink will be systematically summarized.

## 2. 3D Bioprinting Techniques and Bioinks

The successful 3D bioprinting of viable constructs that can structurally and functionally emulate natural tissues/organs highly relies on the adopted bioprinting approaches and appropriate choices of bioink. Owing to the disparate working principles, the application of different 3D bioprinting techniques may lead to distinctive structural features and cell/biomolecule patterning of the constructs. On the other hand, bioink plays a decisive role in the period of both fabrication and in vivo/in vitro application. Suitable properties of bioink can help to support the fabrication process, mediate functions of accommodated cells, and assist in dynamic remodeling. Therefore, it is important to elaborate on the bioink designs anterior to the 3D bioprinting process. This section will summarize the advantages and limitations of three main types of 3D bioprinting techniques, emphasize the key properties of bioinks, and introduce the characteristics as well as the applications of alginate bioink.

### 2.1. Prevalent 3D Bioprinting Techniques 

Different from the traditional 3D printing techniques developed for producing industrial parts [14], 3D bioprinting aims at building biological constructs that could facilitate rehabilitation of injured tissues in vivo or recapitulate physiological functions of natural tissues ex vivo [15]. Such objectives inevitably entail the use of living cells and biomolecules in the fabrication process. These elements are undoubtedly susceptive to harsh manufacturing environments, such as extensive heat, low humidity, and harmful UV exposure, when some traditional 3D printing methods (e.g., selective laser sintering [16], fused deposition modeling [17], stereolithography [18]) are used. Therefore, a variety of 3D bioprinting techniques that enable the deposition of these sensitive blocks has emerged. According to the working principle, the commonly applied 3D bioprinting techniques can be categorized into three sub-types: inkjet-based, microextrusion-based, and light-assisted printing. 

#### 2.1.1. Inkjet-Based 3D Bioprinting Technique

**Figure 2:**
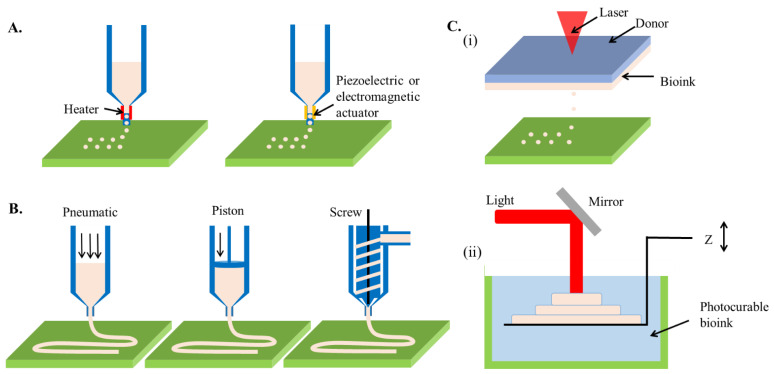
Schematic illustration showing the working principles of prevalent 3D bioprinting techniques. (**A**) inkjet-based 3D bioprinting, (**B**) extrusion-based 3D bioprinting, (**C**) (**i**) laser-assisted jetting, and (**ii**) stereolithography.

Comparable to document printing that ejects a tiny volume of colorful ink onto a paper, inkjet 3D bioprinting selectively deposits bioinks droplet with a volume spanning from nanoliter to microliter toward a substrate [19]. In a layer-by-layer manner, the collected droplets can stack up a three-dimensional construct. The cell/molecule-laden bioink is generally loaded in a cartridge connected to the printing heads of an inkjet printer. As the on-demand printing signals are received, deformations of the printing heads triggered by a mechanical actuator or thermal variation subsequently squeeze the accommodating bioink, resulting in droplet generation (Figure 2A). The deformations of inkjet printer heads are usually driven by a piezoelectric or a thermal unit. While the piezoelectric heads force out the droplets of bioink upon the bending of an electric-sensitive element, the thermal heads impose heat in the affinity of the emission tip to generate a vapor bubble that outputs a bioink droplet. Besides, electrostatic-, electrohydrodynamic-, acoustic-, and microvalve-based modules have been recently used for additive manufacturing [19], capable of intermittently applying pressure to a bioink cartridge, leading to an identical effect of the inkjet-based 3D bioprinting technique. 

It is important to control and stabilize the production of the droplets as they are the units fabricated by the inkjet-based 3D bioprinting technique [20]. Similar to conventional 2D printing, the bioinks can be immediately forced out as a response to the received signals. The frequency of electrical signals applied to induce printing heads deformation mainly dominates the speed of droplets formation. Hence, the rapid and tunable printing speed (1–250,000 droplets per second) is a remarkable merit of this technique [21]. Besides, given the ability to deposit tiny amounts of bioink, inkjet-based 3D bioprinting allows for high fabrication resolution as fine as 2 μm or single-cell dimensions when living cells are encapsulated in bioinks [22]. The size of the ejected droplets depends on the deformation amplitude of printing heads, as well as the viscosity and surface tension of bioinks [REF]. However, to prevent nozzle clotting, the viscosity of bioink is necessarily low (<0.1 Pa·s), which dramatically narrows the range of available biomaterials [23]. In addition, because the density of encapsulated cells is closely related to the viscosity of bioink, it is difficult to print high cell density bioink using the inkjet-based method. The sparsely deposited cells may suffer from apoptosis when the mutual cell interaction is absent, much less forming functional tissue equivalents [24]. 

#### 2.1.2. Microextrusion-Based 3D Bioprinting Technique 

Different from inkjet-based bioprinting that discretely deposits droplets, microextrusion utilizes an extrusion system to continuously squeeze high viscosity bioinks out through a nozzle, forming a fine filament [25]. On control of nozzle movement, the extruded filaments are stacked layer-by-layer to create 3D constructs (Figure 2B). The extrusion system can be either pneumatically or mechanically driven, each of which has distinct advantages. A pneumatic system links stable air pressure provided by a clean compressed air source to the cartridge where bioinks are loaded [26]. Due to the low cost of air source and flexible control of pressure value, the pneumatic system can be easily equipped with an extrusion-based 3D bioprinter. In addition, the pneumatic pressure does not directly contact the bioink, reducing the risks of contamination. However, the pressure loading and residual air in the cartridge might retard the extrusion and overflow of bioinks. In comparison, the mechanical system directly imposes forces to the bioink via a screw or piston module, thus being beneficial for precisely tailoring the extrusion and flow rate of the bioink [27]. Besides, the mechanical force enables the extrusion of bioinks with higher viscosity. However, extensive force may cause detrimental effects on living cells involved in bioink due to the damages to the cell membrane [28]. 

The main advantage of microextrusion-based 3D bioprinting is the ability to use the bioink with a wide range of viscosity (30 mPa·s to >6 ×10^7^ mPa·s) [29]. For this reason, the pool of applicable bioinks is drastically expanded. More importantly, microextrusion permits the direct printing of the bioink with high cell density or even cell spheroid/aggregates, which could facilitate the rapid creation of functional tissues via cell self-assembly [30]. However, during the extrusion process, the cell-laden bioink passes through a sharp nozzle, where the cell viability could be jeopardized by shear stress [31]. Because the gauge of the nozzle is negatively related to the shear stress value, the nozzle size is usually larger than 100 μm to fabricate a highly viable construct [32]. Such a trade-off inevitably results in low printing resolution as the diameter of filaments mainly depends on the nozzle size. 

#### 2.1.3. Light-Assisted 3D Bioprinting Technique

Light can be another power source for driving the 3D bioprinting technique. According to the working principle, the light-assisted 3D bioprinting technique can be further classified as laser-assisted jetting and stereolithography. 

Similar to inkjet-based printing, laser-assisted jetting generates bioink droplets using a laser beam and deposits them onto a platform to construct a 3D objective. Typically, a laser-assisted jetting system is composed of three units, including a light source that focuses a pulsed laser beam, a ribbon consisting of a laser-absorbing layer sandwiched between a transparent donor slide and a bioink layer, and a platform for the collection of droplets (Figure 2C) [33]. When the laser beam is focused on the ribbon, the materials in the absorbance layer (e.g., Au, Ti) react and produce vapor pockets, which subsequently induce droplet formation and jet them toward the platform. The printing results can be manipulated by tuning the parameters of a laser-assisted jetting system, including laser wavelength, pulse energy, signal frequency, the viscosity of bioink layer, and properties of the platform (e.g., the surface tension of substrate and jetting distance) [34]. The use of a pulsed laser can lead to apparent advantages of this technique such as ultrafine resolution at micro-/nano-meter levels and rapid printing speed (5 kHz) [35]. Besides, due to the nozzle-free working principle, laser-assisted jetting is not subjective to cell density and viscosity of bioinks. However, the sophisticated laser source and ribbon composites cause high costs to establish such a bioprinting system. 

Except for the mechanical guiding bioinks to realize their precise deposition, light can also chemically induce selective photo-crosslinking of bioinks to produce 3D complex structures in a layer-by-layer process, namely stereolithography. The first 3D printer was developed based on stereolithography in the 1980s, also called rapid prototyping [36]. It produces parts in a layer-by-layer fashion using photochemical processes by which the selective scanning of laser beams causes crosslinking of photopolymer resin to form 3D solid constructs (Figure 2D). The productive efficiency can be drastically increased with the assistance of a digital micromirror device, which replaces the point-by-point lase scan with matrix projections, namely digital light processing [37]. In addition, based on the mechanism of two-photon polymerization (TPP), the resolution of stereolithography can be reduced to nanometer scales [38]. The advantages of stereolithography are the high resolution (<100 μm) and short printing time (<1 h) [39]. However, the use of ultraviolet (UV) light (100–400 nm wavelength) might induce DNA damage, which triggers apoptosis of cells [40], as well as denaturation of macromolecules [41]. Therefore, despite the long history, stereolithography failed to be involved as an eligible candidate for 3D bioprinting for years. In recent, a variety of innovative photo-initiators emerged (e.g., lithium phenyl-2,4,6-trimethyl-benzoyl phosphinate (LAP) [42], Eosin Y [43], and tris(2,2′-bipyridyl)dichlororuthenium(II) hexahydrate/sodium persulfate (RU/SPS) [44]), which can cause photo-crosslinking of bioinks using visible light, significantly leveraging the strength of stereolithography as a 3D bioprinting technique for building biological constructs. 

### 2.2. Definition and Necessary Properties of Bioink for 3D Bioprinting

According to a putative definition, bioinks can be generally described as ‘a formulation of cells that is suitable to be processed by an automated biofabrication technology that may also contain biologically active components and biomaterials’ [45]. Because cells are a mandatory component, the scope of available biomaterials for formulating a bioink is significantly narrowed. In the field of 3D bioprinting, to accommodate living cells, the bioink should provide a humid and amicable environment, suggesting cell-friendly materials that contain high water contents are the prevailing options for bioinks. Therefore, biocompatible hydrogels, crosslinked hydrophilic biopolymers undissolvable in water, are spotlighted as potential bioinks. In general, both natural polymers derived from creatures (e.g., collagen, gelatin, alginate, fibrin, decellularized extracellular matrix (dECM), and matrigel) or synthetic polymers designed by scientists and engineers (e.g., polyethylene glycol (PEG) and pluronic acid) have been applied in the biomedical field [46]. However, to be a qualified bioink for 3D bioprinting, the biocompatible hydrogel should match a series of requirements. 

#### 2.2.1. Non-Cytotoxicity and Bio-Instructive

As a matrix for building living constructs, the bioink should provide an appropriate microenvironment to the residing cells for supporting their adhesion, proliferation, and migration. The presence of cytotoxic components or compounds can lead to varied cell fates, such as apoptosis, necrosis, and unpredictable cycle arrest [47]. As a result, the fabricated constructs may fail to achieve the purpose of tissue regeneration and function recapitulation. Cytotoxic components could exist on biopolymer chains. For instance, pluronic F127, a pluronic acid, is a commonly used 3D bioprinting material due to its thermal sensitivity and superior printability [48]. However, this material is a surfactant that can lead to membrane disruption of mammalian cells when the concentration is high (>5% (*w*/*w*)) [49]. For this reason, pluronic F127 is considered as a fugitive material for 3D bioprinting of complex structures (e.g., embedded channels, suspended parts) [50,51], rather than a cell-laden bioink. On the other hand, cytotoxic reagents may also be amalgamated during the preparation process. As a representative example, a variety of photo-initiators have been developed to facilitate the photo-crosslinking of bioinks upon different mechanisms (e.g., free-radical chain polymerization, thiol-ene, and photo-mediated redox) [52]. However, most of them are cytotoxic that inevitably impedes cell viability. Therefore, it is critical to consider the choice and concentration of photo-initiators when the light-assisted 3D bioprinting technique and relevant photo-sensitive bioinks are applied.

Beyond protecting cell viability, the bioink is expected to be bio-instructive that can govern cellular activity and functionality. Adhesion is a necessary initial cellular activity for most of the mammalian cells when embedded in bioink hydrogels. Therefore, the bioink should have cell adhesive motifs presenting on the polymer chains so that the cell sense and adhere via ligand-receptor association [53]. A large group of natural ECM proteins contains abundant adhesive moieties for mediating the cell-matrix interaction, while the lack of cell-adhesive property is a general limitation of synthetic polymers [54]. Upon cell adhesion, advanced bioinks are capable of regulating cell functions such as differentiation, alignment, and tissue morphogenesis. The cellular function is commonly mediated by the structural and compositional cues provided by the given microenvironment, such as micro-topological structure, mechanical strength, critical molecule presence, and growth factor gradients [55]. Therefore, efforts have attempted to upgrade biopolymers by either chemical conjugation of biofunctional groups or supplementation with ingredients as reinforcers. In addition to man-designed bioink, the bioink that originated from natural tissue also demonstrated its bio-instructive superiority. For example, the dECM obtained from native tissue can inherit characteristics of native microenvironments in their natural counterparts, and thus the formulated tissue-specific bioinks can significantly promote cell activities and functions [56].

#### 2.2.2. Printability

Printability refers to the ability of a bioink to form and maintain reproducible 3D constructs using a 3D bioprinting technique [57]. In general, printability is associated with the viscosity and the rheological property of a hydrogel material. However, the requirements for these properties significantly vary when different 3D bioprinting techniques are used due to the distinctive working principles.

Viscosity is the resistance of a fluid to the deformation at a given rate. Two main factors affecting the viscosity of a bioink are the molecular weight and concentration of polymers [58]. In general, high molecular weight and concentration result in increased viscosity due to the increased entanglement of polymeric chains. The bioink used for inkjet-based and light-assisted 3D bioprinting should exhibit low viscosity to avoid nozzle blockage or to facilitate the refilling and removal of the unreacted materials. Conversely, the microextrusion-based 3D bioprinting requests a bioink with relatively high viscosity, so that the cell-laden material can be retained statically in the cartridge and acquire improved printing fidelity.

Mathematically, viscosity is defined as the ratio of the shear stress and the shear rate. High viscosity inevitably causes elevated shear stress, which might mechanically damage the encapsulated cells. Therefore, in terms of the microextrusion-based 3D bioprinting technique, the bioink should also exhibit shear-thinning behavior; viscosity falls as the shear rate increases, to reduce the shear stress for protecting the cell viability [59]. Most pre-gel solutions and partially crosslinked hydrogels show the shear-shinning property owing to the polymer disentanglement and molecular orientation along with the shear flow. On the contrary, such a performance is unnecessary when a bioink is adapted to stereolithography because this technique circumvents the use of a nozzle for generating droplets.

Viscoelasticity is another important factor dominating the printability of a bioink. A property of a non-Newtonian fluid displaying viscose flow and elastic shape retention is known as viscoelasticity, governed by the relationship between the storage modulus (G′, energy stored during deformation) and the loss modulus (G″, dissipated by the material) [60]. As the storage modulus surpasses the loss modulus, the bioink is solid-dominant since a large amount of shear-induced energy is elastically stored, leading to elastic shape retention. On contrary, the bioink becomes fluidic because most of the energy is dissipated as heat. The variation of storage and loss modulus is regulated by shear stress. In most cases, both G′ and G″ decrease as shear stress rises, but the G′ falls faster than G″ does, resulting in the viscous flow of bioinks. Such viscoelasticity is especially crucial for conducting microextrusion-based printing. The extrusion process initiates from the flowing of bioink through a nozzle where the material should show a liquid-dominant feature to reduce the shear stress. While the bioink is deposited onto the substrate, the material should preferably convert to a solid-dominant status so that it can resist the deformation to maintain the printed shapes.

#### 2.2.3. Gelation Process

The gelation of bioink plays an important role in 3D bioprinting because it largely determines the result of fabrication, the viability of cells, and the strength of the built constructs. During the gelation process, two key factors, crosslinking condition and gelation speed, should be particularly considered.

Various crosslinking strategies have been developed to induce gelation of bioinks in different mechanisms, including physical crosslinking (ionic interactions, hydrogen bonds, peptide-DNA conjugation, and hybridization), chemical crosslinking (photo-crosslinking, chemical reaction), and enzymatic crosslinking [61]. Despite the multiple selections, the crosslinking condition should be regulated by careful designs of crosslinking parameters to avoid adverse effects on the encapsulated cells or molecules. For example, the concentration and treating time of crosslinkers (e.g., ionic salts and photo-initiators) should be minimized to reduce the adverse effects on cell viability caused by excessive osmotic pressure, cytotoxic effect, and DNA damage. Thermal stimulation ought to be controlled within acceptable ranges of temperature (<37 °C) and period of treatment so that the cell viability and structural stability of molecules can be secured [62].

Upon the printing of droplets or filaments, the flowing bioink should be rapidly solidified to generate firm structures for resisting the gravity-induced material spread and construct collapse. Hence, a high gelation speed of bioink is essential for building a 3D complex structure with high resolution. For instance, collagen is a commonly used bioink that can be thermally crosslinked based on the molecular assembly when incubated at 37 °C [63]. However, the gelation time of thermal crosslinking is too long (>10 min) to maintain the 3D-printed structure, which limits its applications in building complex constructs [64]. In addition, although the gelation kinetics may negligibly influence the printing outcomes when using stereolithography, an increased crosslinking speed could not only accelerate the fabrication process but also improve cell viability by reducing the light irradiation time. Previous studies have reported that the UV exposure time for crosslinking significantly determines the elasticity of hydrogel and the viability of embedded cells [65].

#### 2.2.4. Mechanical Property

Biomechanical signals and the interactions between cells and ECM directly mediate cell fates. As an example, in a 3D culture environment, as the stiffness and strength of bioink are excessively high, cells struggle with the digestion and remodeling surrounding ECMs; therefore, the proliferation and migration of cells are limited [66]. Also, plenty of studies have demonstrated that variation of substrate stiffness can guide the differentiation lineages of stem cells [67]. Besides regulating cellular behaviors, the acquisition of suitable mechanical properties is a prerequisite for successful biomedical applications of 3D bioprinted constructs. The most concerning mechanical properties of a fabricated structure include fracture strength, elasticity, stiffness, toughness, compliance, and fatigue. A biofabricated blood vessel, for instance, needs to possess high ultimate tensile strength (>1 MPa), sufficient burst pressure (>2000 mmHg), and suitable compliance when used as a bypass graft of the coronary artery [68]. Otherwise, the implant is at high risk of graft rupture and intimal hyperplasia. For regenerative implants, the constructs should exhibit comparable mechanical properties to the target tissues, which vary drastically (e.g., the comparison of elastic modulus between adipose and bone [69]. Therefore, the tunable mechanical property is a critical requirement for bioinks. Hydrogel is virtually polymeric chain networks retaining high content of water; the mechanical properties of bioink mainly depend on the density of the chain network, bonding strength of crosslinks, and the mechanism of energy dissipation.

#### 2.2.5. Biodegradability

It is worth noting that under the premise of sufficient incipient mechanical properties, 3D bioprinted constructs should exhibit tunable biodegradation for facilitating the following in vivo and in vitro biomedical applications. First, even entrapped in a hydrogel composed of bio-instructive bioinks, cells cannot always show ideal vitality if biodegradation does not occur. This is because the cells in a 3D microenvironment demand a relatively soft matrix that consecutively undergoes ECM remodeling to support their migration and proliferation [70]. Second, the ultimate objective of regenerative medicine is to repair or replace the injured tissue/organ with artificial implants. Hence, the rate at which degradation/remodeling occurs is important and should ideally be synchronized with the deposition of new cell-derived ECM [71].

The biodegradability of hydrogels is generally based on the hydrolysis of the crosslinks or the polymer backbone [72]. Incorporating cleavable moieties on polymer chains can effectively realize the biodegradation of bioink hydrogel upon ester hydrolysis [73]. Besides, to degrade the polymer backbone of a hydrogel, introducing degradable units into the polymer or involving suitable enzymes are advisable approaches. The main group of enzymes responsible for the degradation of ECM proteins is the matrix metalloproteinases (MMPs) [74]. Because most of the natural polymers originate from ECM proteins (e.g., collagen, elastin, gelatin, and fibrin), the degradation rate of these materials can be easily controlled by promoting or inhibiting the secretion of MMPs from cells following specific signaling pathways.

### 2.3. Alginate as a Bioink

#### 2.3.1. A Brief Overview of Alginate

Alginate is a naturally derived biomaterial extracted from marine algae. Marine algae contain an abundant source of natural polysaccharides and diverse nutrients such as vitamins, salts, iodine, and sterols [75]. Alginate massively exists in the cell wall of algae, the quantification of which depends on the algae species, the type and age of the tissue, and extraction methods. Due to the abundant existence and proficient industrial production, alginate is a low-cost biopolymer.

Alginate is an anionic and hydrophilic polysaccharide naturally derived from brown algae consisting of alternating units, α-*L*-guluronic acid (G), and β-d-mannuronic acid (M), which are covalently linked. The main element in the alginate polymer chain is the carboxylic acid group (COO^−^), which allows for ionic interactions with divalent cations (e.g., Ca^2+^, Sr^2+^, and Ba^2+^), resulting in ionic crosslinking of alginate hydrogel crosslinking. Such an ionic reaction can be immediately triggered upon the presence of divalent cation and preferentially occur at the G-block region. In addition, the G-block is stiffer and more extended in chain configuration than the M-block because of more hindered rotation around the glycosidic linkages [76,77]. Therefore, the biological and physical properties (e.g., biodegradability, viscosity, and mechanical strength) of alginate are basically dependent on its molecular weight, the ratio of M/G, and the distribution of M and G units along the chains. In general, increased molecular weight and concentration of polymer, as well as the fragment of G-block, result in viscose alginate solution or stronger hydrogel, while M-block and MG-blocks enhance the elasticity of alginate [78].

It is known that the positive charges in material might induce an inflammatory response [79]. In this view, as a negatively charged polysaccharide, alginate can support high biocompatibility and cell growth. Following the approval of the U.S. Food and Drug Administration (FDA) [80], the applications of alginate have been extended from a thickening food agent to one of the most important biomaterials for regenerative medicine, nutrition supplements, and drug delivery.

#### 2.3.2. Alginate Bioink

The water solubility as well as rapid and cell-friendly ionic gelation of alginate have attracted scientists to convert this biopolymer to a bioink for 3D bioprinting of living constructs. When exposed to a reagent that contains divalent cations, the diffusion of the ions can induce immediate crosslinking of the 3D bioprinted alginate bioink, leading to the construction of firm structures. Nearly two decades ago, one pioneering study creatively extruded a cell-laden alginate bioink (750,000 human endothelial cells and fibroblasts in 1.5% (*w*/*v*) sodium alginate solution) into a crosslinker bath (5% (*w*/*v*) calcium chloride solution) to fabricate a continuous filament [81]. Using this method, a complex tissue-like construct was 3D bioprinted in a later report [82]. The successful use of alginate as a 3D printable material broadened its biomedical applications, such as tissue regeneration and injectable/localized drug carriers [83].

However, due to the intrinsic limitation of alginate, the constructs built upon the conventional strategies still face several critical challenges. The first obstacle is that, similar to hyaluronic acid or any other unmodified polysaccharide, cell-attachable ligands are not presented in alginate hydrogel. Such an unfavorable microenvironment undoubtedly inhibits cell adhesion and ensuing activity, despite the well-fabricated structures. Therefore, the traditional alginate bioink is commonly used for accommodating anchorage-independent cells, such as chondrocytes which can maintain their phenotype, synthesize and remodel ECM (e.g., proteoglycans and collagens), even without appropriate adhesion [84]. In retrospect, a common application of conventional alginate bioink is the fabrication of chondrocytes-laden constructs for osteochondral tissue engineering. However, when other types of cells are involved, supporting cell adhesion becomes a mandatory feature.

Besides, the enzyme that can cleave the alginate chain is absent in the mammalian body, disabling the regular biodegradation process in vivo. Hence, the degradation of alginate in mammalian bodies usually relies on dissolution as a result of ionic exchanges of divalent cations that crosslink alginate chains with monovalent cation presenting in tissue fluids (e.g., Na^+^ and K^+^) [85]. Such an erratic process causes the degradation rate of alginate-based implants to be uncontrollable.

Moreover, although utilizing a crosslinker bath containing divalent cations is an effective idea to induce desirable gelation and successful 3D bioprinting of alginate, the presence of a high concentration of cations may risk cell viability due to the osmotic shock [86]. In addition, the direct deposition of alginate into solutions may affect the printing solution. Therefore, advanced approaches for improving the printability of alginate bioink are still in demand.

## 3. Strategies for Adopting Alginate as a Bioink

A plethora of innovative methods have emerged to improve the biological activity, tailor the mechanical properties, enhance the printability, and control the biodegradability of conventional alginate bioink. The revolution routes can be generally categorized into (1) the formulation of new bioink systems via physical combination with other components and chemical modification with functional groups; and (2) the innovation of 3D bioprinting strategies.

### 3.1. Formulation of a New Alginate-Based Bioink

#### 3.1.1. Physical Combination with Bioactive Materials

Due to the excellent hydrophilicity, it is possible to mix alginate with a majority of natural and synthetic polymer bioinks. Therefore, blending alginate with other bioactive components that can effectively encourage cellular adhesion to formulate hybrid bioinks is an expedient way to improve the activity of alginate. The co-presenting bioactive polymers in the composite alginate bioink additionally offer a cell-favorable habitat, enabling healthy settle-down of encapsulated cells. So far, a variety of ECM proteins (e.g., collagen [87], gelatin [88], silk fibroin [89,90], matrigel [91], dECM [92], etc.) have been individually or combinatorically incorporated into alginate to develop hybrid bioinks for the applications in 3D bioprinting. In such formulated systems, owing to its tunable viscosity and rapid gelation characteristics, alginate usually plays a role as a solution thickener or structural stabilizer for tailoring the rheology of bioink or facilitating the construction of complex structures. One important consideration when formulating an alginate-based bioink is the balance of biological and physical properties. While an excessively high concentration of alginate inevitably reduces the bioactivity of the involved components, insufficient concentrations may fail to fully utilize the strengths of alginate.

Besides, efforts have attempted to use a second polymer to enhance the mechanical properties of alginate bioink. Forming interpenetrating networks (IPN), referring to polymers synthesized from at least two polymer networks intertwined at the molecular level, is a representative solution [93]. As a pioneering study, Hong et al. combined alginate and poly (ethylene glycol) (PEG) to constitute an IPN hydrogel with both robust strength and biocompatibility, which was applied for 3D bioprinting of living cells [94]. Upon the 3D bioprinting of a construct using the developed bioink, UV irradiation was provided to trigger the photo-crosslinking of the PEG network, followed by the treatment of Ca^2+^ ions to ionically crosslink alginate, forming interlocked polymeric chains. The mechanical reinforcement relies on two mechanisms: the covalently bonded PEG maintains elasticity under server deformation, while the reversible Ca^2+^ crosslinking of alginate dissipates mechanical energy. The printed constructs were able to resist mechanical strength without significant plastic deformation while sustaining high cell viability (75.5 ± 11.6%) over seven days. Despite PEG and alginate supporting cell adhesion, the reported idea opened an avenue for formulating robust and bioactive alginate bioink. Since then, multiple IPN hydrogels such as GelMA/alginate [95,96,97], collagen/alginate [98], and dECM/alginate [99] have been developed for the construction of tissue mimicries. These achievements not only enhance the strength of alginate-based bioink but also yield desirable outcomes of cell activities. Notably, the properties of the resultant IPN hydrogel may depend on the crosslinking sequences of individual monomer participants. For instance, photocrosslinking (e.g., free radical reaction) could be interfered by an ionically crosslinked network formed earlier, resulting in the heterogeneous hydrogel with inferior mechanical strength. Chen et al. formulated an alginate/gelatin IPN hydrogel for 3D cell culture and organ printing. Their outcomes revealed that the altered crosslinking sequences cause different microstructures, leading to varied physiochemical and biochemical properties of the IPN hydrogels. 

The physical participation of suitable additives is also able to enhance the printability of alginate bioink. As a low amount of Ca^2+^ is supplemented into an alginate solution, a pre-crosslink (also called semi-crosslinked) alginate bioink with modulated viscosity, flow behavior, and viscoelastic properties desired for 3D extrusion can be obtained. Falcone et al. investigated the relationship between the concentration of Ca^2+^ and the printability of the pre-crosslinked alginate, demonstrating that within 0.15–0.25 mM of calcium, the bioink showed good extrudability, corresponding to both egg-box dimers and multimers interactions [100]. Interestingly, the source of Ca^2+^ impacts the resultant printability of bioink and mechanical properties of hydrogel due to the varied solubility of cation salts. Kelly et al. studied the printability window of alginate bioinks that were crosslinked by different Ca^2+^ salts (CaCl_2_, CaCO_3_, CaSO_4_) [101]. The low solubility of CaSO_4_ led to slower and more uniform gelation, which improved the mechanical strength of the obtained alginate gel. 

The involvement of light-induced cation generators can enable the biofabrication of alginate bioink using light-assisted 3D bioprinting. Valentin et al. applied 3D stereolithographic printing of alginate hydrogel based on ionic crosslinking by selectively illuminating photoacid generator in the presence of insoluble divalent cation salts. As the spatially provided irradiation of the photoacid generator causes the formation of protons (H^+^), triggering the dissolution of cation salts to generate free divalent cations, which induce the ionic crosslinking of local alginate [102]. In combination with sophisticated laser systems, this method might be used to produce alginate constructs with an ultra-high-resolution (e.g., micro-/nano-meter scale).

To modulate the degradation of alginate bioink hydrogel, one effective way is the involvement of specific enzymes (e.g., the family of alginate lyases) that can specifically recognize the alginate chain and catalyze the cleave the glycosidic bonds [103]. Referring to the molecular configuration of alginate polymer, the choice and concentration of the alginate lyase are key parameters to regulate the alginate hydrogel. Bin et al. applied the 3D bioprinting technique to fabricate biological scaffolds using a dermal-fibroblasts-laden alginate/gelatin bioink [104]. To investigate the degradation property of the constructs, alginate lyase with two different concentrations (0.5 mU mL^−1^ and 5 mU mL^−1^) were incorporated into the bioink. Their results revealed that a higher concentration of alginate lyase leads to faster degradation of alginate. More importantly, the enzyme-governed degradation demonstrated low stiffness and higher porosity, promoting cellular adhesion and proliferation in vitro, as well as facilitating cell infiltration and retention. 

On the other hand, as the dissolution of alginate is generally initiated by ionic exchange, relevant chemicals (e.g., citrate, phosphates, lactates, and Ethylenediaminetetraacetic acid (EDTA)) that help to chelate divalent cation crosslinks are also useful to control the degradation rate of alginate constructs. In a study reported by Wu et al., a 3D bioprinted human corneal epithelial cells (HCECs) laden tissue construct using a collagen/gelatin/alginate composite bioink was cultured in a medium containing sodium citrate to control its degradation rate [105]. The degradation time of the bioprinted constructs can be tailored by altering the mole ratio of sodium citrate/alginate. The tunable degradation dynamics contributed to rapid proliferation and critical gene expression of the embedded HCECs, suggesting the importance of degradation control to cell functionality. However, because ion chelators may also function to inhibit the adhesion between cells and ECMs, the selection and concentration of citrate and EDTA should be carefully considered when using alginate as a bioink to escort living cells.

#### 3.1.2. Chemical Modification with Functional Groups

Diverse types of chemical modifications have been explored to improve the bioactivity of alginate, endowing it with cell adhesion properties. A representative approach is the biofunctionalization of alginate with cell-adhesive peptides. The incorporation of arginine-guanidine-aspartate (RGD), a tripeptide sequence naturally existing in adhesive ECM proteins (e.g., laminin and fibronectin), is a typical method to promote integrin-mediated cell adhesion to alginate. RGD sequences can be recognized by cells via cell-surface integrin receptors, forming focal adhesions [106]. Such associations facilitate cell-ECM interactions and initiate pivotal intracellular signaling cascades for modulating cell adhesion, proliferation, migration, and differentiation. This idea was firstly demonstrated by Rowley et al., in which the alginate was modified with RGD via a carbodiimide chemical reaction [107]. The RGD-conjugated alginate significantly improved the attachment, spread, and differentiation of C2C12 mouse skeletal myoblasts. With the presence of RGD sequences, the 3D bioprinted constructs using the modified alginate bioink can facilitate cell adhesion. Jia et al. built a human adipose-derived stem cells (hADSCs) laden structure by 3D bioprinting of RGD-modified alginate. The hADSCs actively attached and spread in the matrix composed of RGD-modified alginate, while remaining round morphologies in unmodified alginate even after an eight-day incubation period [108].

It is worth noting that, except for RGD, a variety of other peptide sequences can be covalently linked with alginate. While the RGD sequence can be recognized by particular integrins of most cell types, other peptide sequences with better cellular affinity might be desirable when specific cells are used. For instance, Wang et al. modified alginate with arginine-glutamate-aspartate-valine (REDV) peptide sequence that is recognized by α4β1 integrin, particularly expressed by endothelial cells (ECs), to selectively enhance the adhesion of ECs for promoting neovascularization [109]. More importantly, besides improving the bioactivity for cell adhesion, the conjugation with differentiation-inductive peptides can direct specific lineage commitment of encapsulated progenitor or stem cells. In a recent report, Sarker et al. linked RGD and tyrosine-isoleucine-glycine-serine-arginine (YIGSR) peptides, a peptide sequence capable of regulating neuronal differentiation, with a 2% (*w*/*v*) alginate bioink for the 3D bioprinting of neural constructs [110]. After a three-week culture, the bioprinted structures not only facilitate viability and morphology of Schwann cells but also support remarkable directional neurite outgrowth, demonstrating the biofunctionality of the modified alginate bioink. Hence, the conjugation of peptides can be a potential method for converting alginate into a versatile bioink.

Chemical incorporation of photosensitive groups on alginate allows in situ covalent crosslinking under light illumination in the presence of a photo-initiator. The replacement of ionic gelation with the photo-crosslinking strategy is advantageous by offering spatial-temporal control over the gelation process through several configurations, such as the intensity and duration of light exposure, the concentration and the type of photo-initiator, and the extent/pattern of the illumination regions. Notably, the appropriate extent of chemical modification does not necessarily deprive the ionic gelation property of alginate. Therefore, covalent and ionic crosslinking processes can co-present, enabling spatial control over the mechanical property of a 3D bioprinted construct. Samorezov et al. conjugated both methacrylate groups and RGD peptides in alginate and performed varied modification degrees to produce hydrogels that could be either ionically crosslinked, photo-crosslinked, or both (dual crosslinking). By selectively exposing the modified alginate bioink to UV light, the researcher can create patterned structures with regions of dual crosslinked (exposed area) or ionically crosslinked regions (non-exposed area) exhibiting discrepancies in mechanical properties and cell response (i.e., adhesion and spreading) [111]. Moreover, it is known that the loss of crosslinking ions can interfere with the integrity of ionically crosslinked alginate (approximately 40% within 9 days) [112], while the photo-crosslinking of alginate chains can be an effective way to maintain the mechanical and structural stability of the obtained hydrogel due to the presence of covalent bonds. For instance, once the carboxyl of an alginate monomer reacts with 2-aminoethyl methacrylate (AEMA), the methacrylated alginate becomes photocurable, desirably maintaining the mechanical properties of alginate hydrogel and improving its strengths [113].

Enabling the photo-crosslinking of alginate allows for the stereolithography of the relevant bioinks. Ooi et al. used thiol-ene click chemistry to react norbornene-alginate with thiol-containing polymer (e.g., PEG dithiol or 4-arm PEG thiol) crosslinkers, which permitted better spatio-temporal control of alginate rheological and mechanical properties during bioprinting. The norbornene functionalized alginate showed good printability at a lower concentration (2 wt%) and maintained a more stable 3D construct than a printing process which only relies on ionic crosslinking of alginate [114]. 

Besides photo-sensitivity, pioneering studies have attempted to develop thermally-polymerizable alginate, aiming at the creation of versatile alginate bioinks. For example, Wang et al. incorporated glycidyl methacrylate groups in alginate. In the presence of a thermal initiator, the modified alginate could be rapidly (5 to 20 min) crosslinked at 37 °C. These innovations may facilitate the biofabrication process for building alginate constructs, when advanced 3D bioprinting techniques are applied (e.g., heating-assisted extrusion) [115].

With the assistance of chemical modification, the biodegradation of alginate hydrogel can become tunable when degradation cites are incorporated for the association with cell-derived enzymes. A plethora of MMP-sensitive peptide sequences have demonstrated their ability to govern alginate degradation, such as PVGLIG, VPMSMRGG, and GPQGIWGQ. Lueckgen et al. proposed a photo-crosslinkable alginate bioink with the presence of VPMS↓MRGG or GPQG↓IWGQ containing sequences as degradable crosslinkers [116]. The incorporation of these sequences promoted the spread of embedded fibroblasts within these hydrogels whereas the cells remained essentially round in non-degradable counterparts even after 14 days. In vivo outcomes showed higher tissue and cell infiltration into degradable alginate grafts in comparison to non-degradable controls, stressing the importance of involving matrix remodeling cues in the biological performance of bioink constructs. Except for the peptide conjugation, oxidization is also an effective method for offering control over the degradation rate of alginate. It creates hydrolytically labile bonds in the polysaccharide, allowing for easy hydrolysis of alginate backbone [117]. Although the degradation of alginate can be accelerated as the oxidization degree increases, the weakened mechanical property is a vital drawback of the oxidized alginate bioink [118].

Taken together, both physical combination and chemical modification have demonstrated their unique merits for improving the performances of conventional alginate bioink. Meanwhile, the physical and chemical methods can be combined, which can collaboratively provide additional advantages to the resultant bioink. However, each strategy has inevitable drawbacks. While the physical blending usually requests mutual compatibility of the involvements, chemical modification processes might be complicated, which could introduce ingredients harmful to cells. Therefore, the design of bioink is one of the most crucial missions for the 3D bioprinting of alginate hydrogels.

### 3.2. Innovation of 3D Bioprinting Strategy 

In addition to the valuable efforts for bioink modifications, plenty of works have focused on developing novel 3D bioprinting processes that can similarly achieve the precise printing of complex architectures, control of mechanical properties, modulation of degradation, and enhancement of bio-instructive performances.

#### 3.2.1. Aerosol-Assisted 3D Bioprinting

**Figure 3:**
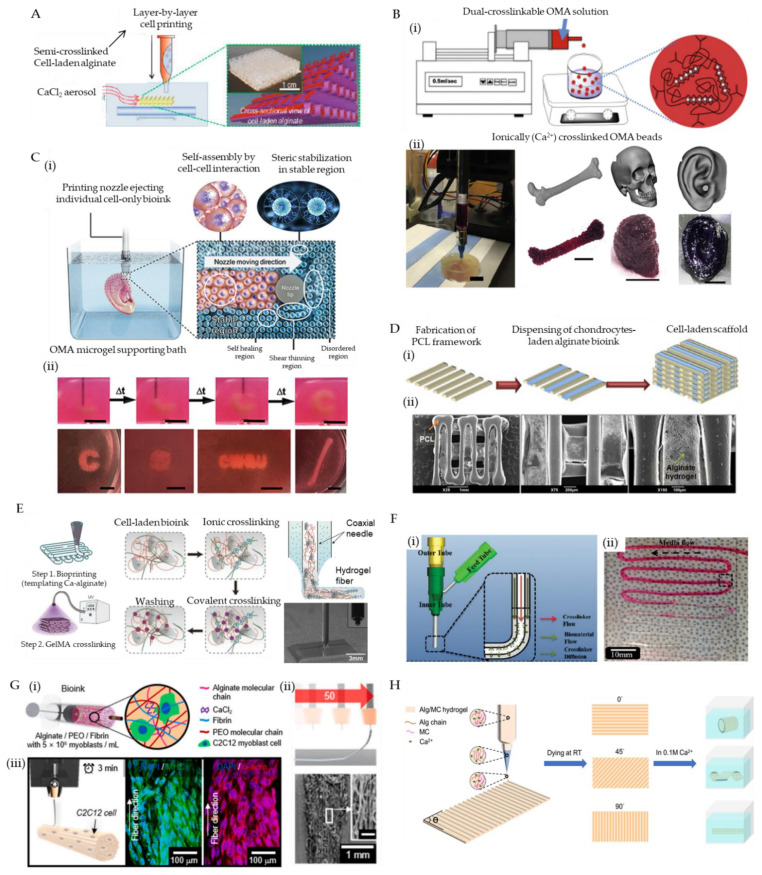
Representative examples that show advanced bioprinting strategies for the adoption of alginate bioinks. (**A**) A schematic of alginate bioink 3D bioprinting assisted by an aerosol-sparing process, producing semi-crosslinked struts (orange: non-crosslinked, purple: crosslinked by exposure to CaCl_2_ aerosol). Reproduced with permission from [119]. (**B**) (**i**) A schematic depicting the fabrication of ionic/covalent dual-crosslinkable OMA beads; and (**ii**) the 3D bioprinting of femur, skull, and ear models using the hMSCs-laden OMA microgel bioinks (scale: femur, 1 cm; skull and ear, 100 μm). Reproduced with permission from [122]. (**C**) (**i**) A schematic of 3D bioprinting of cells within the alginate microgel supporting medium, where the OMA microgels fluidize when stress is applied by the motion of the printing nozzles (shear-thinning region) and rapidly fill in after the needle passes (self-healing region) while the supporting medium without shear presents solid-like properties; and (**ii**) captures of bioprinting a letter “C” (time-course images), a cubic, an acronym “CWRU”, and a femur using stem cell-only bioinks. Reproduced with permission from [123]. (**D**) (**i**) A schematic of the fabrication process of cell-laden PCL/alginate hybrid scaffold using the collaborative 3D bioprinting strategy, and (**ii**) the SEM images of the fabricated porous hybrid scaffold at magnifications of ×25, ×75, and ×150. Reproduced with permission from [124]. (**E**) Schematic illustration of the fabrication process that coaxial extrudes a GelMA/alginate hybrid bioin through the core needle and CaCl_2_ solution through the shell needle, which sequentially undergoes ionic crosslinking and covalent crosslinking, resulting in direct 3D bioprinting of GelMA/alginate fibers. Reproduced with permission from [126]. (**F**) (**i**) A schematic of coaxial printing CaCl_2_/alginate solutions using a core/shell nozzle; and (**ii**) an image of bioprinted alginate microfluidic channels. Reproduced with permission from [128]. (**G**) (**i**) A schematic of the formulated cell-laden alginate/PEO/fibrin bioink for the electrohydrodynamic-direct-writing fabrication, (**ii**) A schematic and SEM images of microfibers fabricated using 50 mm/s nozzle moving speed (scale of the inset: 100 μm), (**iii**) A schematic showing the micro-scale printing of C2C12 cell-laden constructs and immunofluorescent images revealing the orientation of matured muscular fibers. Reproduced with permission from [129]. (**H**) A schematic of 4D bioprinting for fabricate Alg/MC hydrogels and their 3D deformations in CaCl_2_ solution. Reproduced with permission from [130].

To facilitate the direct printing of bioink with improved shape fidelity, aerosols containing gelation initiators can be supplied to create a crosslinking ambiance that can induce rapid gelation of the deposited bioinks. In comparison with the use of an aqueous bath, the aerosolized crosslinking reagents avoid the uncontrollable suspension of printed blocks within the liquid solution due to buoyancy. However, the bioink selected for aerosol treatment should immediately crosslink so that it can be fabricated as stable droplets and firm filaments to ensure printing resolution and enable the construction of complex structures. For this reason, alginate is a good candidate for its rapid gelation feature when encountering divalent cations. Ahn et al. bioprinted a 3D cell-laden scaffold utilizing 3.5% (*w*/*w*) alginate and a cross-linking aerosol produced by fuming 2% (*w*/*w*) calcium chloride solution with an ultrasonic humidifier [119]. In the presence of calcium chloride aerosol, the alginate bioink is directly crosslinked as extruded out from a 310 μm diameter needle, enabling the successful fabrication of a thick construct (4.5 mm height) with a homogeneous pore size (435 ± 32 μm) and fine filaments (355 ± 28 μm) (Figure 3A). The encapsulated pre-osteoblasts (MC3T3-E1) retained high cell viability (85%) after printing, suggesting that the developed fabrication method is unharmful to cells. Notably, the limited diffusion of aerosols can only cause superficial crosslink of the fabricated structures, resulting in weak mechanical properties. Hence, post-treatment such as immersing the printed structure in a Ca^2+^ containing medium is necessary to induce further gelation that can mechanically stabilize the constructs. In this stage, the osmotic shock induced by excessive Ca^2+^ is worth noting.

#### 3.2.2. Microgel-Bioink-Based 3D Bioprinting

Microscale hydrogels (microgels) have been widely used as building blocks for bottom-up tissue engineering. In particular, densely packed or jammed hydrogel microspheres have demonstrated great potential as versatile bioinks for 3D bioprinting [120]. The packed hydrogel microspheres contact and physically trap each other rather than behave as a free suspension in solution, resulting in similar physical properties to bulk hydrogels. Nonetheless, the interactions between the microspheres are weaker than the bonding strength of crosslinked polymer chains, and thus, they can still yield to flow as external forces overcome the inter-microsphere frictions during printing [121]. After printing, the physical associations between microspheres reestablish, which can support the fabricated structures. Such a shear-thinning and self-healing behavior enables microgel to be a suitable form of bioink for 3D bioprinting techniques.

Jeon et al. reported controlled assembly of cell-laden dual-crosslinkable alginate microgels composed of oxidized and methacrylated alginate (OMA) [122]. The human bone marrow-derived mesenchymal stem cells (hMSCs)-laden OMA microspheres (300 μm) were firstly produced using a coaxial airflow-induced microgel generator that forms OMA monomers (2.5% (*w*/*v*)) directly into ionic crosslinking solution (0.2 M CaCl_2_). The packed cell-encapsulated OMA microspheres exhibited shear-thinning behavior, shear-yielding feature, and self-healing property, which enables the 3D extrusion of diverse customized constructs (Figure 3B). After the flexible assembly of OMA microspheres based on fabrication processes, low-level UV illumination (20 mW/cm^2^) was supplied to initiate photo-crosslinking, which tightly locked the adjacent microspheres via covalent bonds, forming stabilized structures.

In addition to bioink, packed microgels can also be utilized as a support medium for 3D bioprinting. The support medium is fluidized under low shear stress, allowing motion of a depositing needle within the bulk consisting of microgels. As the shear stress caused by needle movement is removed, the locally fluidized microgel reservoir rapidly self-heals, forming a stable medium that firmly holds the printed bioinks. The same group expanded the OMA microgels as a photocurable support medium to directly print individual cell-only bioink [123]. The rheological characteristics of the OMA microgel bath enabled the high-resolution deposition, positioning, and structuring of hMSCs without creating crevasses. The ensuing photo-crosslinking enhanced the structural and mechanical integrity of the OMA microgel bath, providing a long-term stable culture and differentiation condition to the printed cellular constructs (Figure 3C). Dissociation of the photocured microgel bath by gentle agitation can enable the acquisition of matured 3D tissue analogs. Besides, the idea of ‘in-bath printing’ is also useful to enable the direct printing of soft alginate solutions. In a representative report, Hinton et al. defined a novel 3D bioprinting technique named freeform reversible embedding of suspended hydrogel (FRESH), which permits the printing of soft bioinks (alginate, fibrin, collagen, and matrigel) within a second gelatin microgel supportive bath [123]. The reversible thermal-gelation of gelatin enables the creation of gelatin microgel at a low temperature that facilitates the suspension of bioink and the removal of gelatin bath as the temperature rises (incubating at 37 °C), helping to separate the printed constructs.

#### 3.2.3. Collaborative 3D Bioprinting 

The integration of multiple printing heads achieves the simultaneous dispensing of diverse bioinks in a single fabrication step. When a wide range of bioinks and biomaterials are applied, collaborative 3D bioprinting is helpful to meet various needs, including robust mechanical property, acceptable printability, and tunable degradability.

Using a multi-head deposition system (MHDS) equipped with four microextrusion dispensers, polycaprolactone (PCL) and human chondrocytes-encapsulated alginate bioink (4% (*w*/*v*)) was co-printed to fabricate a cell-laden scaffold for cartilage regeneration [124]. During printing, PCL was firstly melted and printed as a framework followed by subsequent deposition of cell-laden alginate bioink in the spaces between the lines of PCL layers. Such a procedure was repeated, resulting in the stacking up of a 3D PCL/alginate heterogeneous structure (Figure 3D). After treating 100 mM CaCl_2_, the crosslinked alginate offered a cell-friendly microenvironment for the loaded chondrocytes, while the PCL framework not only allowed the precise positioning of soft alginate bioink but also provided adequate mechanical strength to the biological construct for resisting the load-bearing condition in vivo. Using an identical approach, alginate bioink carrying human mesenchymal stromal cells was also used to 3D bio-print multiple layered constructs for osteochondral tissue regeneration [125].

In contrast to the parallel organization of nozzles for sequential printing, multiple needles can be assembled coaxially that allow the simultaneous extrusion of different bioinks. Given the advantages of rapid ionic gelation, alginate is an optimal bioink for such a coaxial bioprinting technique. When alginate is co-dispensed with a crosslinker (e.g., CaCl_2_ solution) through a pair of coaxially configurated nozzles, instant crosslinking can be induced, forming firm alginate fibers, which enhances its printability. Colosi et al. used a core/shell nozzle to produce a blended GelMA (4.5% *w*/*v*) and alginate (4% *w*/*v*) bioink in the core and 0.3 M CaCl_2_ solution in the shell [126]. When the core and shell materials came into contact at the outlet of the coupled nozzles, the alginate in the bioink was ionically gelled by exposure to Ca^2+^ ions, producing robust 150 μm wide filaments and providing excellent printability for stacking a thick structure (1 mm height) (Figure 3E). The GelMA was subsequently cross-linked by UV treatment, and the alginate gradually dissolved within culture media after 10 days. Owing to the bioactivity of GelMA, the embedded human umbilical vein endothelial cells (HUVECs) showed improved cell migration and organization.

Another fascinating merit of coaxial bioprinting lies in its ability to build tubular constructs. By increasing the number of needles, multiple cell-laden alginate-based bioinks can be concentrically layered, resulting in biomimetic cannular tissue mimicries (e.g., blood vessel and urethra) [127]. In a pioneering study, the group of Ozbolat developed a novel printable vessel-like microfluidic channel fabrication method that enables the direct bioprinting of cellular microfluidic conduits [128]. Alginate and chitosan bioinks were applied to construct both freestanding and bulk-hydrogel embedding channels to test their nutrition-transporting functionality through perfusion of cell-type oxygenized media, demonstrating the great promise of the microfluidic channels for the development of vascular networks (Figure 3F).

#### 3.2.4. Micro-/Nano-Scale 3D Bioprinting

To better emulate native tissues, there is an increasing requirement to build structures with ultrafine resolutions, which can further recapitulate the hierarchical structure and composition of the native ECM. Such replications of the cellular microenvironment may suggest new opportunities for the generation of functional tissue and organs. For instance, cell-cell interactions occur at the micro- to nanometer scale [129]. In addition, mechanical property and topological structure cues provided by fibrillar ECM components are important modulators of cell fates [130]. Therefore, micro-/nano-scale 3D bioprinting of bioinks is spotlighted as a new direction to improve the bio-instructive performances of constructs through the micro-structural definition.

Among all types of abovementioned 3D bioprinting approaches, only the TPP technique can achieve a resolution finer than dozens of micrometers. Reports from different groups have demonstrated that the TPP technique allows for reaching a structure resolution less than 100 nm [131]. As a bioactive bioink that can undergo photo-crosslinking, GelMA has been applied for TPP in several studies [132,133,134]. Technically, any photocurable bioink is adaptable to the TPP technique. Therefore, although there are no relevant reports, the formulated alginate bioinks (e.g., chemically methacrylated alginate) can be utilized for the 3D bioprinting of micro-/nano-scale structures using the TPP technique.

Electrospinning is another fabrication method that can obtain nanofibers resembling the natural ECM. However, owing to the rigid chain conformation and limited chain entanglement, the alginate, a natural polyelectrolytic polymer, does not readily electrospin [135]. Typically, flexible and uncharged synthetic polymers (e.g., polyethylene oxide (PEO) and polyvinyl alcohol (PVA) can be supplemented to improve the spinnability of alginate [136]. Due to the formation of hydrogen bonds between alginate and these polymers, the repulsive force among polyanionic molecules is drastically decreased to facilitate the chain entanglement, which permits the production of nanofibers. Notably, despite the given high-intensity electrical field, studies have demonstrated that the electrospinning of biocompatible hydrogels is compatible with the processing of living cells if the electric voltage is controlled in an appropriate range, even achieving the reconstruction of functional constructs embedding cardiomyocytes or neural cells [137,138].

However, one drawback of electrospinning is the difficulty in spatially organizing the produced fibers. To overcome this challenge, electrohydrodynamic and electrowriting techniques that can utilize the flexibility of the 3D bioprinting technique were developed to align the fibers, achieving direct fabrications of anisotropic tissue mimicries. In a recent work reported by Yeo et al. a fully patterned microfibrous construct fabricated using fibrin-assisted alginate bioink encapsulating myoblasts/endothelial cells and a novel electrohydrodynamic-direct-writing approach was proposed for skeletal muscle tissue engineering [139] (Figure 3G). This new biofabrication method helped to provide mechanically stable and topographical cues to the involved cells, inducing rapid and efficient regeneration of skeletal muscle tissues.

Despite the currently available techniques for 3D bioprinting of fine structures, the remaining challenges are the unstable mechanical and swelling properties of hydrogel, which might cause damage and morphing of the fabricated construct. Consequently, to tackle this issue, strategies that are useful to increase the stiffness of the hydrogels include varying the degree of crosslinking, grafting of a secondary robust material, and the formation of interpenetrating networks.

#### 3.2.5. 4D Bioprinting of Alginate-Based Bioink

The recently emerged concept of 4D bioprinting refers to the morphing of a 3D printed structure in response to an environmental stimulus with the fourth dimension being time-dependent changes. Therefore, the fabrication of a 4D bioprinted construct is a type of programmable matter that govern shape transformation resulting from the reaction of the printed products with relevant parameters within an environment (e.g., temperature, PH, humidity, magnetism, electricity, light, etc.) or cellular activity over time (e.g., cell contraction, cell migration, etc.) [140]. A large amount of stimulus-responsive materials has been developed for smart autonomous robotics, biomedical devices, drug delivery, and tissue engineering [141].

A central idea for the 4D bioprinting of hydrogel bioinks as shape-morphing materials is the exploitation of their flexible swelling/shrinking behavior. By controlling the swelling/shrinking rates or degrees, the volume of the hydrogel can be altered sizably and anisotropically, enabling the building of transformable hydrogel constructs. Due to the reversible ionic crosslinking of alginate as a function of involvement and chelation of divalent cations, an alginate architecture with gradient crosslinking density can be dynamically modulated under specific stimulations [142]. Hence, it is possible to utilize alginate as a smart bioink for 4D bioprinting. In a pioneering work, Kirillova et al. utilized a visible-light-curable methacrylated alginate bioink to 3D bioprinted cell-laden biological films [143]. Upon photo-crosslinking under green light and mild drying, the films immersed into solutions (e.g., water, PBS, and cell culture media) instantly fold into tubes due to the swelling of alginate hydrogel. When exposed to calcium salt or EDTA solutions, the ionic crosslinking/de-crosslinking of alginate can harness its de-swelling/re-swelling performances, thereby fulfilling the reversible shape transformation of the films.

However, such dynamic shape transformation is quite limited, alone with its poor printability; thus, pure alginate bioink for 4D bioprinting has been rarely reported. Despite the limitations, when mixed with other biopolymers such as methylcellulose and polydopamine, alginate-based bioink can obtain the desired shape-morphing ability. In a recent study, alginate and methylcellulose were combined to formulate highly printable and shape morphing hydrogels with excellent rheological properties, extrudability, and shape fidelity of the printed structures [144] (Figure 3H). In another report, alginate/polydopamine bioink was developed for 4D printing of artificial tissues [145]. Continuous efforts for formulating alginate with other materials might lead to the production of new advances for diverse biomedical applications.

In conclusion, the advanced material formulation and novel biofabrication approach allowed the appearance of desired features in conventional alginate bioinks. With improved versatility and controllability, these innovative strategies could extensively leverage the strength of the 3D bioprinting technique in biomedical applications, which will be focused on in the next section.

## 4. Applications of Alginate-Based Bioink

### 4.1. Regenerative Implants

With the assistance of advanced strategies, diverse functional artificial tissue constructs have been 3D bioprinted along with living cells for the regeneration of cartilage, bone, skin, blood vessel, muscle, heart valve, neuron, etc.

#### 4.1.1. Osteochondral Tissue

Cartilage and bone are typically hard tissues that bear extreme stresses during body movement. Since these tissues are frequently exposed to dynamic loads (e.g., compression, tension, bending, etc.), the grafts must provide adequate and stable mechanical/structural support and sufficient inorganic ECMs to realize the healing of relevant tissue defects [146]. Notably, although various material formulations and fabrication methods have been developed to match the stringent requirements for the regeneration of hard tissues, these approaches are generally not applicable to carrying living cells. Hence, it is unsuitable to categorize these achievements into the group of bioink and 3D bioprinting.

When alginate bioinks are applied for constructing osteochondral tissues, the limited mechanical strength of hydrogel is the first challenge. Using the collaborative printing strategy, the chondrocytes-laden bioinks can be co-deposited in a mechanically robust thermoplastic scaffold, achieving the requirements for both mechanical properties and bioactivity [147]. Kundu et al. co-printed PCL struct and alginate bioink carrying chondrocytes layer-by-layer to build cartilage structures that can be implanted into the bear-loading condition in a rabbit model. With the addition of TGF-β, the graft showed a great cartilage-like extra cellular matrix formation [124]. Similarly, Lee et al. demonstrated that the substantial improvement of mechanical strength (tensile modulus from 2.5 MPa of pure alginate scaffolds to 15.4 MPa of PCL/alginate hybrid constructs) was attributed to the adhesion between PCL structs [148]. However, despite biocompatible thermoplastic polymers such as PCL, PLGA, PLA, and PU being able to assist alginate scaffolds to meet the requirement of mechanical properties, the degradation rate of these materials should be carefully considered so that the implants could endure loads during a long-term period of bone regeneration.

Upon physical combination with other components or chemical modification, advanced alginate bioinks have proven their advantages for the fabrication of cartilage and bone tissue constructs. Luo et al. optimized the printability of gelatin-alginate bioink by mixing cellulose nanofibers, resulting in superior rheological performances and printability [149]. The developed bioink not only allows high-precision bioprinting of a specific-designed meniscal prototype but also achieves long-term cellular viability and acceptable ECM accumulation of encapsulated fibrochondrocytes. When mixed with highly bioactive materials, the cytocompatibility of alginate-based bioink can be significantly improved. Yang et al. combined alginate and collagen to formulate a hybrid bioink for 3D bioprinting, which can effectively maintain the phenotype of chondrocytes and promote the expression of cartilage-specific genes [87]. Kelly and colleagues added porcine articular cartilage extracellular matrix (0.2 or 0.4% *w*/*v*) into alginate solution (2.45% *w*/*v*) to obtain a bioink for 3D bioprinting of hMSCs [150]. To improve the printability of the hybrid bioink, 0.018 M solution of CaCl_2_ was added to induce the pre-crosslinking. Upon the construction of a 3D construct, the bioink was completely crosslinked in a 0.06 M CaCl_2_ bath for 20 min. Their results suggested that the presence of cartilage-tissue-specific ECM can significantly enhance chondrogenesis by guiding the activity of embedded stem cells. 

Bioactive ceramics can effectively improve the regeneration of bone tissue. Hydroxyapatite (HA) and calcium phosphate (CP) are calcium phosphate compounds commonly used for bone tissue repair. These biocompatible inorganic materials have negligible cytotoxicity when blended into alginate hydrogel with an optimal concentration, which can effectively improve the biological performances of alginate-based bioink. In an investigation conducted by the group of Hofmann, gelatin, alginate and hydroxyapatite (HA) were hybridized to obtain a novel hydrogel composite for bone 3D printing [151]. The thermo-sensitivity of gelatin and the chemical crosslinking of alginate helped to achieve rapid gelation and long-term structural integrity of the 3D-printed constructs. The embedded hMSCs survived during the printing process and showed high cell viability (over 85%) after three days of subsequent in vitro culture. CP has been also used to improve the mechanical properties and osteoconductivity of 3D bioprinting hydrogel constructs.

Relevant osteogenic or chondrogenic molecules and growth factors are also useful to enhance the biological functions of the bioprinting constructs, However, the release kinetic of carried payloads must be carefully designed. In general, the consecutive bio-instructive function originating from growth factors is desired during a long-term regeneration period. Hence, a sustained release profile is necessary. In a representative study conducted by Park et al., osteoblasts and bone morphogenetic protein 2 (BMP-2) were co-encapsulated in an alginate/alginate-sulfate bioink, demonstrating the promotion of osteogenesis by the prolonged BMP-2 activity in the designed composite bioink [152]. 

Although these novel approaches have enabled the 3D bioprinting of cell-laden alginate-based constructs for the regeneration of osteochondral tissues, it still faces unmet challenges related to biological and mechanical properties. Further efforts should continuously contribute to explore advanced strategies to extend alginate-based orthopedic applications.

#### 4.1.2. Skin Tissue

In comparison with hard tissues, as the hydrogel material is originally soft and fragile, the cell-laden alginate-based bioink show a greater premise for engineering soft tissues, such as skin, blood vessel, muscle, etc. Human skin has three layers consisting of epidermis, dermis, and hypodermis, each of which is composed of different cell types. In addition to the clear stratification, multiple appendages (e.g., hair folic, sweat glands, vasculature, etc.) are present in the limit space of skin (0.5–4 mm thickness) [153]. Although skin has greater self-regeneration capacity than most tissues after being injured, the healing of large-scale or deep wounds is usually limited, suffering from scar formation. Therefore, to 3D bio-print this complex and multi-layered architecture to assist in wound healing, bioinks should show superior printability and biological performances.

To enhance the printability and bioactivity of alginate bioinks, honey and gelatin have been incorporated. Honey is a readily available natural material, known for its role in wound healing and skin tissue regeneration. Datta et al. demonstrated that the involvement of honey in varying concentrations (1–5%) can effectively modulate the viscosity and printability of 5% alginate bioink [154]. In addition, the presence of honey is also conducive to the improvement of proliferation when 3T3 fibroblasts are encapsulated and 3D bioprinted.

Liu et al. blended gelatin and alginate to compose a bioink with improved printability [155]. Upon the results of rheological evaluation, 2% alginate and 15% gelatin were selected as an optimal formulation, which can help acquire complex constructs (e.g., nose and ear) with high printing resolution (151 ± 13.04 μm) in a low temperature (4 °C) chamber due to the presence of gelatin. The ionic gelation of alginate components after treating 2% CaCl_2_ further stabilized the fabricated structures. Using this hybrid bioink, human amniotic epithelial cells (hAECs) and Wharton’s jelly-derived mesenchymal stem cells (WJMSCs) were encapsulated to bio-print a skin-biomimetic bi-layered membranous construct. The outcomes of in the vitro study indicated that the hAECs displayed a superior epithelial cell phenotype, while WJMCSs showed great angiogenic potential and fibroblast phenotype.

Beyond the successful fabrication of skin tissue mimicries, mechanical properties should be also considered to match that of natural skin for better clinical handling and wound-healing effect. Shi et al. modulated the mechanical strength of alginate/gelatin bioink using a three-step crosslinking strategy [156]. Gelatin components in the 3D bioprinted structure were first physically crosslinked at 4 °C for 30 min, followed by the ionic gelation of alginate by immersing in 1% CaCl_2_ solution for 1 h. Eventually, the construct was further treated with 1% EDC and 0.25% NHS for 1 h at 4 °C to induce covalent crosslinking of gelatin. Through such a series of crosslinking courses, dermal substitutes with physicochemical properties that match human skin tissue can be created. However, despite the reported high proliferation rate of seed cells being able to demonstrate the biocompatibility of the printed structure, such a process (e.g., low-temperature incubation, chemicals exposure) is inevitably harmful to cells, and thus not applicable for printing living cells.

#### 4.1.3. Vascular Tissue

Blood vessels, especially artery and vein, contain three concentric layers—a monolayer of endothelium tissue in contact with the bloodstream, a dense layer containing circumferentially aligned smooth muscle cells, and a layer of connective tissue in the abluminal side [156]. Each layer involves specific cell types that undertake distinctive missions. For this reason, an ideal blood vessel graft should compartmentally possess all these elements to fully recapitulate vascular functions.

The emergence of the coaxial bioprinting strategy paves a way for the convenient fabrication of tubular constructs. Due to rapid gelation, alginate has been considered as an optimal bioink for this approach. Zhang et al. coaxially bioprinted perfusable vascular conduits using primary human umbilical vein smooth muscle cells- (HUVSMCs) laden alginate bioinks with different concentrations (3–5% wt) [157]. The dimension of the printed tubes (inner diameter and wall thickness) are tunable by printing diameters. Gao et al. revealed that the sizes of the bioprinted hollow tubes depend on the gauge of the inner needle as well as the flow rate and concentration of alginate bioink and crosslinkers (CaCl_2_), demonstrating that coaxial bioprinting is a flexible technique for producing conduits [158].

On the other hand, the hydrogel-made vascular tissue constructs might have limited mechanical strength and stability to be used as a graft and in vitro tissue-modeling platform. To overcome this challenge, additional strategies can be integrated. For example, Jia et al. fabricated HUVECs- and hMSCs-laden bioink consisting of alginate, 4-arm poly(ethylene glycol)-tetra-acrylate (PEGTA), and GelMA pre-polymer solutions [159]. Upon a UV illumination procedure, the PEGTA and GelMA were covalently crosslinked, which provided a secondary polymeric network forming IPN hydrogel, significantly enhancing the mechanical properties of the bioprinted vascular tissue constructs. In addition, the blended bioink was proven to support the spreading and proliferation of embedded cells due to the presence of bioactive GelMA.

As exhibiting physiological functions (e.g., anti-thrombosis, responsive contraction and relaxation) are critical for ensuring the safety of vascular grafts, the bioink should show superior bioactivity to guide cells activities and form relevant functional tissues. In this regard, the involvement of bioactive components is an effective manner. In a recent report, small-diameter blood vessel grafts comprising compartmentally organized endothelial cells and muscle cells were successfully 3D bioprinted using a bioink that is composed of alginate and VdECM [160]. The application of a triple-layered coaxial nozzle helps to produce a small diameter tube (approximately 2 mm) consisting of a thin HUVEC layer (50 μm) surrounded by a thicker HAoSMCs layer (800–1000 μm). The prematured construct under a customized dynamic culture condition showed uniaxial and circumferential alignment of HUVECs and HAoSMCs, respectively, emulating that of the natural counterparts. Meanwhile, the massive accumulations of de novo human ECM proteins (collagen and elastin) were found to help reinforce the mechanical strengths (burst pressure and ultimate tensile strength). After being implanted into the abdominal aorta of the rat for three weeks, the grafts showed muscular layer remodeling and integration into the host tissue, as well as good patency and preservation of intact human endothelium tissues.

Collectively, the ultimate objective of tissue engineering is to build functional constructs that regenerate or replace the damaged tissue or entire organ. Recent efforts regarding 3D bioprinting of alginate-based bioinks have been used to establish a variety of regenerative tissue implants. Except for the cases reviewed above, representative studies contributed to constructing other types of tissues are summarized (Table 1) to provide relevant information and comparisons.

### 4.2. In Vitro Tissue Modeling

Upon a period of cultivation and stimulation, even under an in vitro condition, the cells residing in bioinks can populate, migrate, and self-assemble, possibly resulting in maturation and functionalization of targeted tissue/organ. For this reason, except when regarding as tissue substitutes, a variety of 3D bioprinted alginate-based constructs can be also used as in vitro tissue/organ equivalents, since these models can resemble the complex anatomical and pathological features of the emulated targets, including skin, kidney tubules, blood vessels, cornea, and skeletal muscle. Relevant reports have been comprehensively summarised with insightful comments elsewhere, which can provide an overview of this point [180,181].

Besides the tissue/organ analogs, advanced in vitro tissue models, organoids and organ-on-a-chip, emerged in recent years for convenient modeling of physiological process and disease pathology, as well as evaluating drug safety and therapeutic effects. Given the high flexibility, the 3D bioprinting technique has been translated to establish a variety of diagnostic and monitoring platforms. Although in its infancy, alginate-based bioink has demonstrated its advantages for achieving this objective.

#### 4.2.1. 3D Bioprinting of Alginate-Based Organoids

Organoids, defined as miniature organs, are generally developed by the 3D culture of mammalian stem/progenitor cells or embryonic stem cells in the presence of necessary physiological cues and matrices. The formation of organotypic constructs relies on cell proliferation, self-organization, and differentiation into specific lineages, whereby multiple types of cells resembling both the architectural and functional features of the target organ can be generated [182,183].

Despite the 3D bioprinting of organoids still being at an incipient stage, undifferentiated pluripotent stem cells or differentiated stem cells have been 3D bioprinted using alginate-based bioinks to generate organoids in vitro. For instance, Gu et al. extruded human iPSCs within a hybrid bioink composed of alginate, carboxymethyl-chitosan, and agarose, differentiated in situ to self-assemble 3D embryoid bodies expressing three germ markers [184]. The study demonstrated that the 3D bioprinting process has no adverse impacts on the pluripotency and differentiation lineage of stem cells.

The formulation details of a bioink play a pivotal role not only in maintaining cell viability but also in offering stem cell niches for directing cell fates. It is worth noting that unexpected adhesion to ECM may adversely affect the self-assembly of stem cells, hindering the formation of organoids. Resultingly, the non-adhesive property of alginate can be useful for organoid construction. In a recent report, Nguyen et al. compared the activity of human-induced pluripotent stem cells (hiPSCs) co-cultured with chondrocytes in bioinks consisting of nanofibrillated cellulose (NFC) with alginate or hyaluronic acid [164]. While the hiPSCs sustained pluripotency and formed cartilaginous tissue in NFC/alginate bioink after five weeks, the regressed proliferation and pluripotency were found among the cells encapsulated in NFC/hyaluronic acid. Hence, the selection of bioink materials is critical for the 3D bioprinting of organoids. In another work, Capeling et al. demonstrated that alginate supports the growth of human intestinal organoids (HIOs) in vitro and leads to HIO epithelial differentiation virtually indistinguishable from Matrigel-grown HIOs [185]. Their findings indicated that purely mechanical support from alginate might be sufficient to promote the survival and development of specific organoids. Due to the low cost and easy-to-use advantages of alginate, novel and cost-effective manners for organoid construction can be available.

To date, diverse types of human organoids, such as brain, retina, lung, liver, kidney, pancreas, stomach, and intestine, have been established to study infectious diseases, genetic disorders, and cancers [186]. In a representative study, Yang et al. micro-extruded HepaRG cells (a differentiated hepatic cell line) embedded in a bioink formulated from alginate and gelatin to 3D bioprinting hepatorganoids [187]. The organoids not only demonstrated the detoxification function of the liver in vitro but also facilitate liver injury when implanted into a mouse model.

Drug screening and discovery is another potential application of organoids. However, for industrial use, the platform must be high-throughput (10,000–100,000 compounds tests per day) to ensure the efficiency and repeatability of investigations [188]. 3D bioprinting, as an automated biofabrication technique, can construct massive and reproducible organoids. Lawlor et al. replaced the manual production of kidney organoids with automatic bioprinting of iPSCs into multi-well plates, which improved throughput, quality control, scale, and structure [189]. Although no report has used the 3D bioprinting technique to produce alginate-based organoids on a large-scale, recent attempts have demonstrated that microfluidic devices can produce massive alginate droplets which encapsulate tumor pieces, resulting in the generation of mammary tumor organoids for high-throughput drug screening [190]. Considering the similar working principle of some advanced fabrication strategies (e.g., coaxial extrusion, core-shell inkjet-based printing), such results are possibly achievable by 3D bioprinting techniques.

#### 4.2.2. 3D Bioprinted Alginate-Based Organ-on-a-Chip

Compared with organoids, the organ-on-a-chip (OoC), generally defined as a microfluidic cell culture device, includes microchannels in well-organized multiple types of cells. The presence of microchannels allows both the control of nutrient delivery and the manipulation of biomechanical stimulus (e.g., stress, flow, cyclic motion) or biochemical microenvironments (e.g., oxygen gradients and chemotaxis) [191]. When 3D bioprinting is applied to establish an OoC platform, one essential goal is the incorporation of microfluidic channels within organized living constructs to mimic the dynamic conditions in the human body.

Using a classical approach, the reversible ionic crosslinking feature permits the use of alginate as a sacrificial bioink to create microchannels in bulk hydrogels. For instance, interconnected 3D vascular networks in hydrogels can be obtained after the removal of a previously embedded alginate lattice template upon the treatment of EDTA solution. The ensuing seeding of HUVECs on the luminal wall could lead to the development of a vasculature-on-a-chip [192,193]. The construction of a complex template requires great printability of alginate, which can be tackled by relevant strategies discussed in an earlier section. However, the indirect fabrication process usually requires cell-seeding as an additional procedure to achieve vascular function, which could result in low efficacy or deficient endothelium formation. In past years, this methodology has been widely utilized to construct diverse OoCs that reflect the pathophysiology of blood vessels, vascularized tissues, tissue interfaces, and disordered microenvironments (e.g., inflammation and cancer).

Stereolithography can directly build microfluidic devices immediately. Due to the high resolution of the laser source, the printing precision is markedly better than that produced by the template-sacrificing method. Upon specific chemical modifications (e.g., methacrylation), the photocurable alginate is certainly adaptable to this strategy for building an OoC. Surprisingly, through physical combination with photoacid generators and divalent cation salts, the stereolithographic printing of ionically-crosslinked alginate OoC has also been achieved by a research group of Wong [102]. The ironical crosslinking does not deprive the dissolubility of the alginate hydrogel, which contributes to direct collective cell migration from different initial geometries, revealing differences in front speed and leader cell formation.

As an advanced strategy, coaxial bioprinting techniques can directly fabricate freestanding and perfusable tubes by co-dispensing alginate bioinks and crosslinkers containing Ca^2+^ ions through a core/shell nozzle. However, because alginate lacks cell-adhesive moieties, bioactive materials are usually supplemented to improve their cell-friendly performance. Gao et al. proposed a hybrid bioink composed of VdECM and alginate. By coaxially printing it with pluronic F127 accommodating Ca^2+^, vasculature-on-a-chip and atherosclerosis-on-a-chip capable of recapitulating the physiological functions and inflammatory responses of vascular tissues were successfully constructed [194,195]. Similarly, Singh et al. developed an advanced renal-tubule-on-a-chip using a triple coaxial nozzle [196]. By simultaneous or individual switch-on the middle and shell nozzles, a renal tube with either monolayer or dual layers can be fabricated, enabling the construction of a vascularized renal proximal tubule on a chip-like device. In another study, bioprinted blood and lymphatic vessel pair were achieved by Cao et al. using an identical strategy, which was applied as a tumor-on-a-chip system to evaluate transportation mechanisms of pharmaceutical compounds inside the tumor microenvironment [197]. Notably, despite the eye-catching achievements, a main drawback of the coaxial bioprinting strategy lies in its inability to fabricate branched channels, and thus significantly impedes its applications in engineering an OoC with complex patterns of microchannels.

## 5. Conclusions and Future Perspectives

Due to non-bioactivity, poor printability, uncontrollable biodegradability, and unstable structural/mechanical stability, it is difficult to regard alginate as an acceptable bioink for 3D bioprinting of biological constructs. To overcome these limitations, diverse advanced strategies have been proposed in recent years, relying on either the reformulation of bioink recipes (e.g., physical blending and chemical modification) or the innovation of the biofabrication process (e.g., aerosol assistance, microgel bioink, collaborative printing, micro-/nano-scale printing, and 4D bioprinting). Employing these solutions, the applications of alginate have been drastically extended to construct a variety of tissue-equivalent for tissue regeneration and repair, as well as novel in vitro platforms (organoids and organ-on-a-chip) that facilitate the interpretation of disease pathophysiology and screening of drug safety/efficacies.

Given the current achievements summarized in this article, future efforts that continuously dive into innovative strategies would keep harvesting much more promising results. For instance, an interesting work has demonstrated the possibility of in situ 3D bioprinting of skin tissue using a handheld skin printer [198]. The rapid crosslinking merit of alginate is a key prerequisite for the direct printing of cell-laden bioinks onto wound regions. Their proof-of-concept verifications for the formation of biomaterial sheets in murine and porcine excisional wound models suggest the capacity of printing-assisted healing for wounds that are subject to respiratory motion. This attempt may strengthen the bridge between fundamental research of 3D bioprinting and clinical applications. 

Besides, the freestanding and biofunctional vasculatures can be continuously fabricated using the coaxial bioprinting strategy at present. Using this method, it might be possible to create a circulatory system in vitro, which can structurally and biologically connect multiple individual tissue/organ-on-a-chip to obtain an organ-cluster-on-a-chip (e.g., a digestion system consisting of stomach, intestine, liver, and kidney chip modules) or even human-on-a-chip platform.

On the other hand, several strategies might be still in their infancy, and thus no relevant reports are provided in the current review. However, the great potential of these innovative ideas might bring great revolutions. One intriguing work is the 4D bioprinting technique. The human body is a highly dynamic environment entailing various physiological processes (e.g., a beating heart and intestine peristaltic movement). Growing awareness has justified that the recapitulation of such dynamic physical features play an important role in tissue and organ morphogenesis. Therefore, exploring the shape-morphing ability of the 4D bioprinted constructs can be an advisable approach for emulating the physiological dynamics of organs (e.g., periodical and consecutive motions) in fabricated tissue constructs to provide cells with a stimulative environment that is analogous to their natural counterparts.

## Figures and Tables

**Table 1:** Representative cases of 3D bioprinting regenerative implants using alginate-based bioinks.

Tissue	Bioink	Cell Type	Strategy	Achievement	Ref.
**Cartilage**	Nanocellulose-alginate bioink	Human nasoseptal chondrocytes	Physical combination	Constructs with high fidelity and stability	[161]
	hyaluronic acid/alginate bioink, PCL as a scaffold	Human articular chondrocytes	Physical combination	Improved printability, gelling abilities, stiffness and degradability	[162]
	Alginate Sulfate–Nanocellulose Bioinks	Bovine chondrocytes	Chemical modification	High shape fidelity, good printability	[163]
	Nanocellulose/Alginate Bioink	iPSCs	Physical combination	Bioprinted iPSCs for cartilage regeneration	[164]
	Collagen-alginate bioink	Rat chondrocytes	Physical combination	Improved mechanical strength, enhanced cells adhesion, proliferation	[87]
	Polylactic Acid (PLA) Nanofiber−Alginate Hydrogel Bioink	Human adipose-derived stem cells	Physical combination	Improved hASC metabolic activity and proliferation	[165]
	Alginate, gelatin, and fibrinogen as bioink	hMSCs	Physical combination	The addition of TGF-β1 and BMP-2 promoted cells differentiation	[166]
	Alginate and short sub-micron polylactide (PLA) fibers	Human chondrocytes	Physical combination	High cell viability	[167]
**Bone**	alginate-sulfate bioink	MC3T3-E1 osteoblasts	Chemical modification	Improved osteoblastic proliferation and differentiation	[152]
	Graphene oxide/alginate bioink	hMSCs	Physical combination	Enhanced osteogenic differentiation, improved printability	[168]
	Alginate CaCl_2_ bioink	Human bone marrow-derived MSCs	Chemical modification	Increased osteogenic differentiation	[169]
	RGD-γ-irradiated alginate and nano-hydroxyapatite (nHA) complexed to plasmid DNA (pDNA)	Human bone marrow-derived MSCs	Chemical modification	Superior levels of vascularization and mineralization	[170]
**Vessel**	Sodium alginate Fibroblasts	L929 mouse fibroblasts	Collaborative 3D bioprinting	Multilevel fluidic channels	[171]
	Sodium alginate, collagen	HUVECs	Microgel-bioink-based 3D bioprinting	Achieved rapid and efficient in vivo angiogenesis.	[172]
	VdECM/alginate bioink	HUVEC/HAoSMCs	Collaborative 3D bioprinting	As transplants in vivo for three weeks	[160]
	gelatin-based alginate/carbon nanotubes blend bioink	Fibroblasts	Physical combination	Enhanced mechanical properties	[173]
	Gelatin-methacryloyl (GelMA) + PEGDA + alginate lyase	Vascular smooth muscle cells/vascular endothelial cells	Collaborative 3D bioprinting & Physical combination	Two-cell-layered structure	[174]
**Skin**	Gelatin and sodium alginate hydrogel, fibroblast cells	Fibroblasts	Physical combination	Situ 3D bioprinting	[175]
	Sodium alginate, sodium carboxymethyl cellulose	/	Physical combination	Repaired rabbit wound defeat	[176]
**Nerve scaffold**	Sodium alginate, gelatin	Rat Schwann cells	Physical combination	Improved cell adhesion and related factor expression, in vivo	[177]
**Muscle**	Gelatin Methacryloyl-Alginate Bioinks	Mouse C2C12 myoblast cells	Collaborative 3D bioprinting	Dually crosslinking can provide the optimal niche for muscle tissue formation	[178]
	PEG-Fibrinogen (PF)/alginate	Human C2C12 myoblast cells	Collaborative 3D bioprinting	Formed multinucleated myotubes	[179]
